# Epigenetic Analysis through MSAP-NGS Coupled Technology: The Case Study of White Poplar Monoclonal Populations/Stands

**DOI:** 10.3390/ijms21197393

**Published:** 2020-10-07

**Authors:** Francesco Guarino, Berthold Heinze, Stefano Castiglione, Angela Cicatelli

**Affiliations:** 1 Department of Chemistry and Biology “A. Zambelli”, University of Salerno, via Giovanni Paolo II 132, 84084 Fisciano (SA), Italy; fguarino@unisa.it (F.G.); acicatelli@unisa.it (A.C.); 2 Department of Forest Genetics, Austrian Federal Research Centre for Forests, 1131 Vienna, Austria; berthold.heinze@bfw.gv.at

**Keywords:** MSAP, NGS, poplar, epigenetic, methylation

## Abstract

Over the last several decades, several lines of evidence have shown that epigenetic modifications modulate phenotype and mediate an organism’s response to environmental stimuli. Plant DNA is normally highly methylated, although notable differences exist between species. Many biomolecular techniques based on PCR have been developed to analyse DNA methylation status, however a qualitative leap was made with the advent of next-generation sequencing (NGS). In the case of large, repetitive, or not-yet-sequenced genomes characterised by a high level of DNA methylation, the NGS analysis of bisulphite pre-treated DNA is expensive and time consuming, and moreover, in some cases data analysis is a major challenge. Methylation-sensitive amplification polymorphism (MSAP) analysis is a highly effective method to study DNA methylation. The method is based on the comparison of double DNA digestion profiles (*Eco*RI-*Hpa*II and *Eco*RI-*Msp*I) to reveal methylation pattern variations. These are often attributable to pedoclimatic and stress conditions which affect all organisms during their lifetime. In our study, five white poplar (*Populus alba* L.) specimens were collected from different monoclonal stands in the Maltese archipelago, and their DNA was processed by means of an innovative approach where MSAP analysis was followed by NGS. This allowed us to identify genes that were differentially methylated among the different specimens and link them to specific biochemical pathways. Many differentially methylated genes were found to encode transfer RNAs (tRNAs) related to photosynthesis or light reaction pathways. Our results clearly demonstrate that this combinatorial method is suitable for epigenetic studies of unsequenced genomes like *P. alba* (at the time of study), and to identify epigenetic variations related to stress, probably caused by different and changing pedoclimatic conditions, to which the poplar stands have been exposed.

## 1. Introduction

In the last twenty years, many studies have focused on epigenetic modifications in plants, particularly on DNA methylation status, and on the mechanisms driven by external stimuli (abiotic stresses, pedoclimatic conditions, climate changes etc.) that are exploited by plants to counteract mid- and/or long-term changes, with or without the involvement of signalling molecules (e.g., jasmonic acid, salicylic acid and reactive oxygen species).

In plants, DNA methylation commonly occurs within three main sequences: CG, CHG and CHH (where H is A, C or T); however, the symmetrical CpG and CpA(T)pG sites are reported to be the most frequent targets of cytosine modification. Changes in DNA methylation contribute to the transcriptional silencing of transposable elements or foreign DNA, maintaining genome stability against non-homologous recombination, regulating the transcription of genes [1,2,3] and to DNA repair during replication.

It has been shown that DNA methylation can be temporary and/or meiotically/mitotically transmitted to the progeny and maintained in plant memory, even when the causative stress is removed. Plant DNA is usually highly methylated (6–30%), however, large differences among species have been observed [4].

Changes in DNA methylation are also crucial for promoting phenotypic variation in living organisms [5]. Some authors observed epigenetic polymorphisms among different varieties of rice, and found that, on the basis of the methylation status of the genes among the individuals analysed, the polymorphisms led—in some cases—to novel phenotypes [6]. In addition, several studies have investigated the possibility that DNA methylation status might better reveal the genetic biodiversity within or among populations [7,8,9], especially in species that spread through natural vegetative propagation. In fact, in this case, epigenetic modifications may compensate for reduced genetic biodiversity, counteract pedoclimatic variations and present an additional source of phenotypic variation. This aspect has been investigated recently in two natural plant species that are widely distributed—namely, the white poplar (*Populus alba* L., a dicot species) [8] and the giant reed (*Arundo donax* L., a monocot species) [9]. In both cases, the natural populations inhabited environments that were characterised by different pedoclimatic conditions, forming large monoclonal stands, but at the same time, their DNA methylation profiles revealed several differences specific to their geographical location, suggesting that epigenetic diversity should be considered in the diversity analyses that are commonly employed in the framework of the Convention on Biological Diversity. Moreover, it has been demonstrated that epigenetic modifications are hereditable [10], and this supports the result obtained by Raj et al. (2011) [11], who found that the clones with the most divergent transcriptomes and clone history had the most marked differences in the extent of total DNA methylation, suggesting an epigenomic basis for the clone-history-dependent transcriptome divergence.

The majority of the epigenetic studies of plants have been carried out using methylation-sensitive amplified polymorphism (MSAP) analysis. This method is based on two parallel double enzymatic digestions of DNA, with *Eco*RI combined with two isoschizomers which are differently sensitive to DNA methylation: *Msp*I and *Hpa*II. The comparison of the two MSAP profiles provides information about the DNA methylation status of the samples [12]. The popularity of the method, particularly for those species for which the genome sequence is not available, is due to its reliability, reproducibility and low cost. However, a limitation of the method is related to the lack of knowledge of DNA traits showing different methylation status. Here we explored whether next-generation sequencing (NGS) combined with MSAP analysis could be used to overcome this limitation, with the aim of identifying regions of the genome that are differentially methylated. At the present, only two studies have reported the combined use of MSAP analysis and NGS in plants, but their results were limited to the identification of polymorphic amplicons, without the determination of the DNA methylation status of the sequenced fragments or the identification of the pathways that were enriched for differentially methylated sequences. In other studies, some authors applied bisulphite NGS sequencing after MSAP [13], particularly when the genome sequence was known, as in the case of rice as an alternative, they proceeded with amplicon electrophoresis on polyacrylamide gels and the isolation of polymorphic bands, then sequenced through Sanger technology [14]. Our study promises to make a step forward in this kind of molecular analysis, providing a new protocol of coupled MSAP-NGS and a new flowchart for data mining, useful for obtaining information about the methylation status of the sequenced fragments and their involvement in specific genetic pathways and biological processes. This new combined method is particularly suitable for species for which the reference genome is not yet available, which was the case for *P. alba* at the time when this study was undertaken. To test this approach, we analysed five trees of a clone of white poplar, collected in the Maltese archipelago and already genetically analysed [15]. Previous studies have demonstrated that white poplar populations are constituted by large monoclonal stands [16,17], as commonly reported for poplars of the *Populus* section [18], and many other species which adopt vegetative propagation as a prevalent way of reproduction [19]. Therefore, the white poplar represented an excellent species to test the MSAP-NGS method, due to the incomplete knowledge of its large and complex genome.

## 2. Results

### 2.1. Technical Overview of MSAP-NGS Methodology

In order to exploit the power of the MSAP protocol for obtaining information about the DNA methylation status of the monoclonal stands of white poplar on the island of Malta, we combined it with NGS, which provided the nucleotide sequences of the MSAP amplicons obtained by PCR. The combined MSAP-NGS procedure required several steps (see below) and specific biostatistics data analyses.

**Figure 1:**
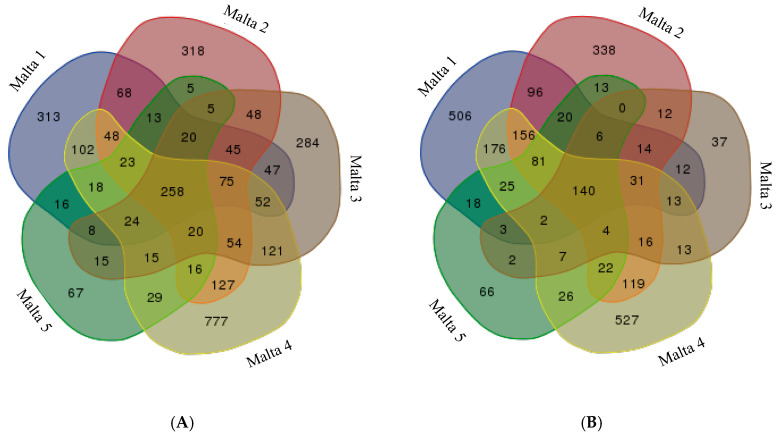
Comparison among the five white poplar samples of the contigs obtained from (**A**) *Eco*RI-*Msp*I digestion (3031); (**B**) from *Eco*RI-*Hpa*II digestion (2501).

Step 1: The reads obtained by DNA NGS amplicons, obtained from each single digestion (*Eco*RI-*Msp*I or *Eco*RI-*Hpa*II dataset) of all the samples, were used to assemble de novo a reference genome constituted by 5532 contigs. The contigs obtained from the mapping of reads to the reference genome were analysed in order to assay the reproducibility of the analyses and to verify the number of contigs obtained for each sample. Two Venn diagrams were elaborated comparing contigs obtained from each sample and each enzyme (Figure 1).

The number of contigs obtained by read mapping of fragments of both digestions was comparable (Figure 1); when a single dataset was considered (*Eco*RI-*Msp*I, or *Eco*RI-*Hpa*II digestion), different white poplar samples shared a high number of contigs; moreover, each sample also showed private contigs. Comparing the data, the magnitude of the number of private contigs was similar in the two different datasets (*Msp*I or *Hpa*II digestions). In particular, sample Malta 5 showed the lowest number of private contigs but with the same magnitude for both datasets (67 for *Msp*I and 66 for *Hpa*II digestion), whilst Malta 4 had the highest one (777 in the case of *Msp*I, and 527 in the case of *Hpa*II). For a better understanding of the molecular background of the epigenetic changes, we considered the most relevant contigs to be those that consistently showed identical polymorphisms among samples.

Step 2: In order to detect differentially methylated sequences or known genes (Archive S1A), contigs of each sample and of each digestion were analysed using BLASTN GenBank/NCBI, adopting *A. thaliana* as the reference species.

**Figure 2:**
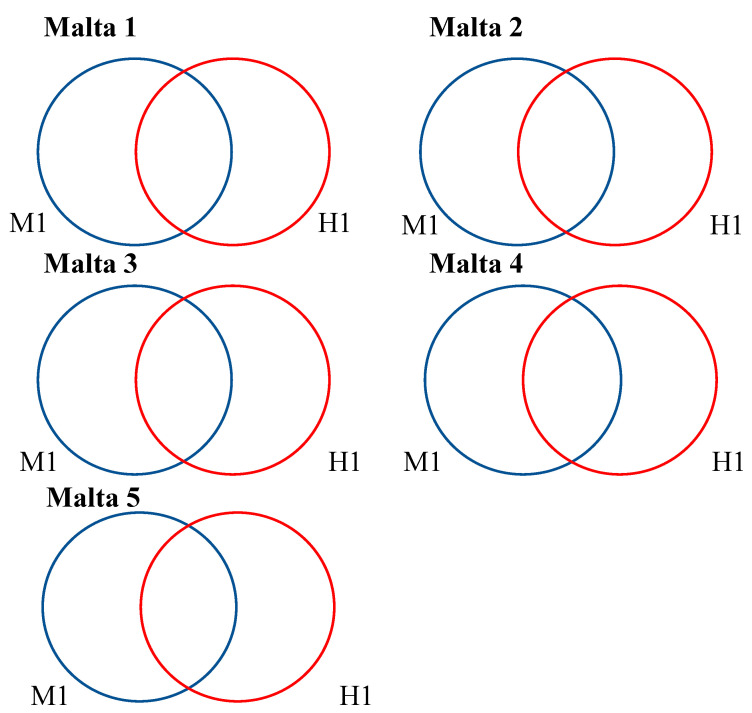
Comparison between the *Eco*RI-*Msp*I and *Eco*RI-*Hpa*II profiles of each sample. The numbers represent the genes identified from the contigs vs. *A. thaliana.* The list of private or shared genes is reported in Archive S1B.

Step 3: In order to estimate the DNA methylation status of the genes identified (on the basis of the principles that we adopted and described above) for each white poplar sample, a Venn diagram was singularly elaborated including genes derived from either *Eco*RI-*Msp*I or *Eco*RI-*Hpa*II datasets (Figure 2).

The results reported in Figure 2 show that the magnitude of gene numbers obtained from each digestion was comparable among all the samples. In particular, the number of genes unaffected by DNA methylation (shared genes between *Msp*I and *Hpa*II datasets—M1-H1, Figure 2) ranged between 33 (Malta 5) and 60 (Malta 4); in the case of genes affected by double-strand methylation of inner cytosine or hemi-methylation of inner cytosine (genes amplified just after the *Msp*I–M1-H0) the number was between 22 and 41, whilst, when the hemi-methylated CHG-sites (hemi-methylation of inner and outer cytosine—M0-H1) were considered, the number of genes ranged between 29 and 67. A list of the genes affected by different DNA methylation status is reported in Table S1A.

Step 4: On the basis of the BLASTN and Venn analyses results, a pathway analysis was performed (for each sample and for each DNA methylation status) to identify those pathways enriched for genes showing different DNA methylation status. In addition, genes underwent gene ontology analysis in order to identify the enriched pathways, including genes affected by diverse DNA methylation status.

**Figure 3:**
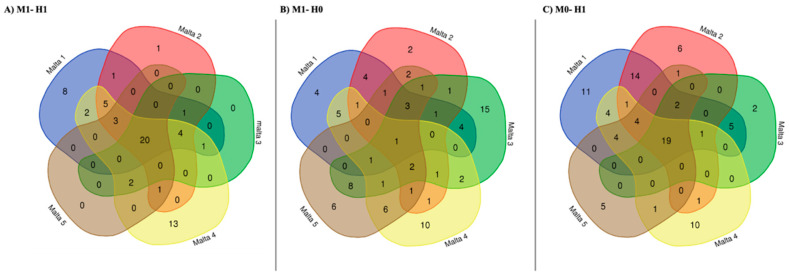
Comparison among the genes found in all five samples with the same DNA methylation status (previously estimated in Figure 1). In particular, (**A**) reports the results of the comparison of the genes identified in each sample for the M1-H1 condition, (**B**) those for M1-H0 condition, and (**C**) those in M0-H1 condition. The list of shared or private genes is reported in Table S1C.

Step 5: Another step of the data analysis aimed to identify genes with the same DNA methylation status, specific to each sample or shared among the five white poplar samples. Toward this purpose, a Venn analysis was elaborated on differently methylated fragments (Figure 3).

### 2.2. Gene Ontology Analyses

Malta 1

**Figure 4:**
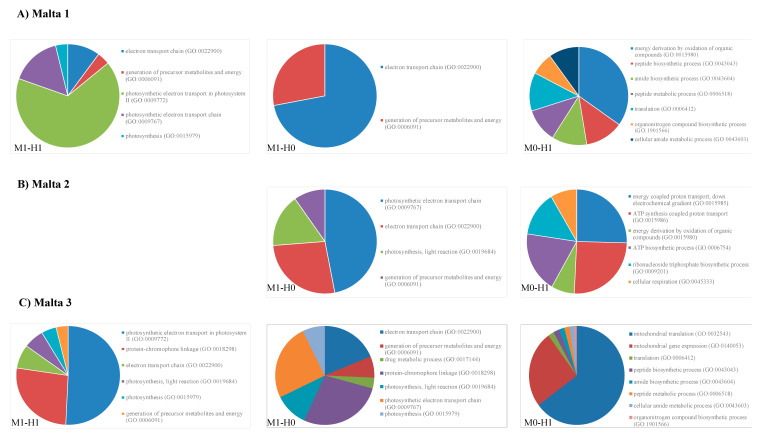

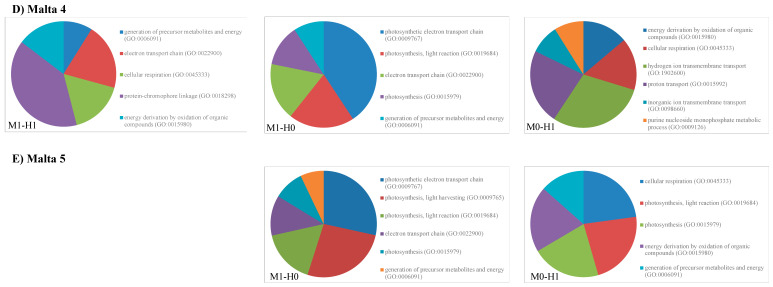
Biological processes affected by the diverse DNA methylation status in Malta 1 (**A**), Malta 2 (**B**), Malta 3 (**C**), Malta 4 (**D**) and Malta 5 (**E**).

For sample Malta 1, different biological processes were affected by the diverse DNA methylation status (Figure 4A). Table S1C reports only the results with false discovery rate < 0.05.

In Table S1B (no methylation), the lowest *p*-value (10^−7^) was associated with photosynthetic electron transport in photosystem II (GO:0009772), electron transport chain (GO:0022900) and generation of precursor metabolites and energy (GO:0006091), and photosynthetic electron transport chain (GO:0009767; FDR *p*-value 10^−5^). An intermediate *p*-value (10^−5^) was observed for photosynthesis (GO:0015979), whilst the lowest *p*-value (10^−3^) was observed for protein-chromophore linkage (GO:0018298), cellular respiration (GO:0045333), photosynthesis, light reaction (GO:0019684), energy derivation by oxidation of organic compounds (GO:0015980) and oxidation-reduction process (GO:0055114).

In the case of condition M1-H0 (double-strand methylation of inner cytosine or hemi-methylation of inner cytosine), the genes affected were only over-represented with a significant *p*-value (*p* < 0.05) in the pathway of the generation of precursor metabolites and energy (GO:0006091).

The genes affected by M0-H1 hemi-methylated CHG-sites (hemi-methylation of inner and outer cytosine, Table S1B) were involved in the following pathways with a significant *p*-value (FDR < 0.05): heme transport (GO:0015886), mitochondrial translation (GO:0032543), iron coordination entity transport (GO:1901678), mitochondrial gene expression (GO:0140053), cytochrome complex assembly (GO:0017004), cellular respiration (GO:0045333), energy derivation by oxidation of organic compounds (GO:0015980), generation of precursor metabolites and energy (GO:0006091), translation (GO:0006412), peptide biosynthetic process (GO:0043043), amide biosynthetic process (GO:0043604), peptide metabolic process (GO:0006518), cellular amide metabolic process (GO:0043603), organo-nitrogen compound biosynthetic process (GO:1901566), oxidation-reduction process (GO:0055114) and cellular nitrogen compound biosynthetic process (GO:0044271).

Malta 2

The GO analysis of the sample Malta 2 (Figure 4B) revealed that in the case of “no methylation condition”, no pathway was significantly enriched (Table S1B).

In the case of M1-H0 (double-strand methylation of inner cytosine or hemi-methylation of inner cytosine), four pathways were significantly overrepresented (FDR *p*-value < 0.05): photosynthetic electron transport chain (GO:0009767), electron transport chain (GO:0022900), photosynthesis, light reaction (GO:0019684) and generation of precursor metabolites and energy (GO:0006091).

When the condition M0-H1 hemi-methylated CHG-sites (hemi-methylation of inner and outer cytosine) was considered, the number of overrepresented pathways significantly increased, particularly those related to ATP-synthesis-coupled proton transport (GO:0015986), energy-coupled proton transport, down electrochemical gradient (GO:0015985) and ATP biosynthetic process (GO:0006754).

Malta 3

The results of the analyses of Malta 3 reported in Figure 4C (Table S1B) revealed that, in the case of “no methylation condition”, the most overrepresented pathways were protein-chromophore linkage (GO:0018298; FDR *p*-value 10^−5^), photosynthesis (GO:0015979) and generation of precursor metabolites and energy (GO:0006091; *p*-value 10^−3^). In the case of the condition M1-H0 (double-strand methylation of inner cytosine, or hemi-methylation of inner cytosine), the most over-represented pathways were: electron transport chain (GO:0022900), generation of precursor metabolites and energy (GO:0006091; FDR *p*-value 10^−8^), protein-chromophore linkage (GO:0018298), photosynthesis, light reaction (GO:0019684; FDR *p*-value 10^−5^), ATP metabolic process (GO:0046034), photosynthetic electron transport in photosystem II (GO:0009772), purine ribonucleoside triphosphate metabolic process (GO:0009205), purine nucleoside triphosphate metabolic process (GO:0009144) and others, while in the case of M0-H1 hemi-methylated CHG-sites (hemi-methylation of inner and outer cytosine) the most represented pathways were: peptide biosynthetic process (GO:0043043), amide biosynthetic process (GO:0043604), peptide metabolic process (GO:0006518) and cellular amide metabolic process (GO:0043603; FDR *p*-value *p* 10^−4^).

Malta 4

In the case of sample Malta 4 (Table S1B), GO analyses (Figure 4D) showed that when no methylation condition was considered, the most over-represented pathways were: generation of precursor metabolites and energy (GO:0006091; FDR *p*-value 10^−4^), protein-chromophore linkage (GO:0018298), electron transport chain (GO:0022900), cellular respiration (GO:0045333), energy derivation by oxidation of organic compounds (GO:0015980; FDR *p*-value 10^−3^), photosynthesis (GO:0015979) and photosynthetic electron transport in photosystem II (GO:0009772; FDR *p*-value < 0.05). In the case of condition M1-H0 (double-strand methylation of inner cytosine or hemi-methylation of inner cytosine) the pathways most represented were: photosynthetic electron transport chain (GO:0009767), photosynthesis (GO:0015979) and generation of precursor metabolites and energy (GO:0006091) that showed a *p*-value equal to 10^−8^. The other pathways related to photosynthetic electron transport chain (GO:0009767) and electron transport chain (GO:0022900) showed *p*-values of 10^−7^ and 10^−5^, respectively. In the case of M0-H1 hemi-methylated CHG-sites (hemi-methylation of inner and outer cytosine) we found the greatest number of over-represented pathways, and in particular the most represented were: hydrogen ion transmembrane transport (GO:1902600), proton transport (GO:0015992), cellular respiration (GO:0045333) and energy derivation by oxidation of organic compounds (GO:0015980; FDR *p*-value 10^−5^).

Malta 5

The sample Malta 5 showed that, in the case of the no-methylation condition, there were no pathways significantly enriched (Table S1B). When the condition M1-H0 (double-strand methylation of inner cytosine or hemi-methylation of inner cytosine) was considered, the most enriched pathways were: generation of precursor metabolites and energy (GO:0006091) and photosynthesis (GO:0015979) (FDR *p*-value 10^−4^) and photosynthetic electron transport chain (GO:0009767) and photosynthesis, light harvesting (GO:0009765; FDR *p*-value 10^−3^).

In the case of M0-H1 hemi-methylated CHG-sites (hemi-methylation of inner and outer cytosine) the two most represented pathway were: photosynthesis (GO:0015979; FDR *p*-value 10^−5^) and generation of precursor metabolites and energy (GO:0006091; FDR *p*-value 10^−4^).

### 2.3. Venn Analysis of Differently Methylated Fragments

The results of the Venn analyses showed that 20 genes unaffected by DNA methylation (Figure 3A) were shared by all five samples. The shared genes were not significantly enriched for any biological process. However, among them, four encode transfer RNAs (tRNAs; Figure S1B).

Whilst eight, one and thirteen specific genes were identified in Malta 1, Malta3 and Malta 4, respectively, in contrast, the eight genes specific to Malta 1 were significantly enriched for the biological processes related to response to virus (*p* < 0.005) and to heat (*p* < 0.05). The unique gene specific of Malta 3, PSI P700 apoprotein A2 (psaB) was not suitable for an enrichment analyses, whilst the 13 identified as specific to Malta 5 were enriched for biological processes related to mitochondrial translation and mitochondrial gene expression (*p* < 0.0005).

**Figure 5:**
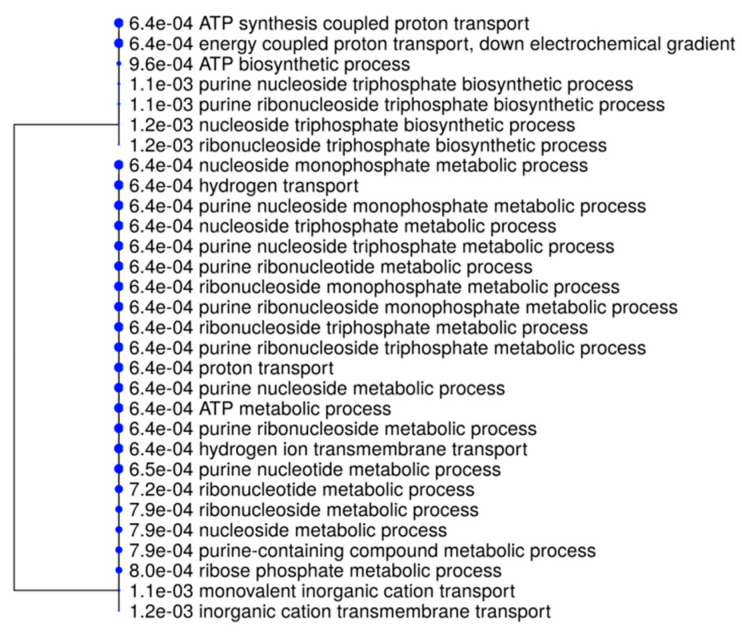
A hierarchical clustering tree. Pathways with many shared genes are clustered together. Larger dots indicate more significant *p*-values.

In the case of the double-strand methylation of inner cytosine or hemi-methylation of inner cytosine (Figure 3B), only a single gene was shared among all five samples, identified as cytochrome b6/f complex subunit 4 (petD), whilst several genes were specific for Malta 1 (4 genes), Malta 2 (3 genes), Malta 3 (15 genes), Malta 4 (10 genes) and Malta 5 (6 genes). However, as in the case of Malta 3 and 5, the genes identified, which were affected by double-strand methylation of inner cytosine or hemi-methylation of inner cytosine, were significantly enriched (*p* < 0.05) for some biological processes—mainly those related to metabolic processes. These processes are reported in Figure 5.

**Figure 6:**
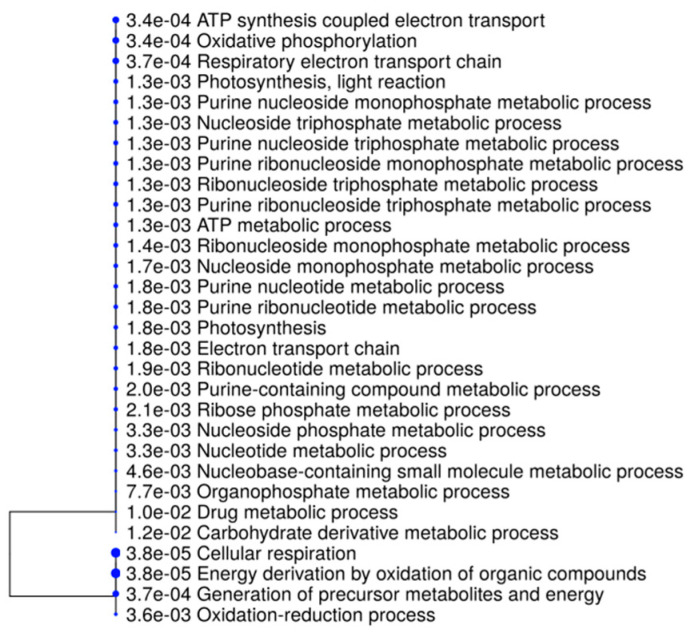
A hierarchical clustering tree. Pathways with many shared genes are clustered together. Larger dots indicate more significant *p*-values.

In the case of Malta 5, the biological processes affected by this kind of methylation and with highly significant *p*-values were related to cellular respiration or energy derivation by the oxidation of organic compounds (Figure 6).

In the case of hemi-methylated CHG-sites (hemi-methylation of inner and outer cytosine; Figure 4C), the number of genes shared among all the samples was 19, whilst those specific for Malta 1, Malta 2, Malta 3, Malta 4 and Malta 5 were eleven, six, two, ten and five, respectively. The genes characterized by hemi-methylated CHG-sites (hemi-methylation of inner and outer cytosine) and shared among samples were not significantly enriched for any biological process, but many of them were recognized as tRNA—more than that expected (Figure S1C).

**Figure 7:**
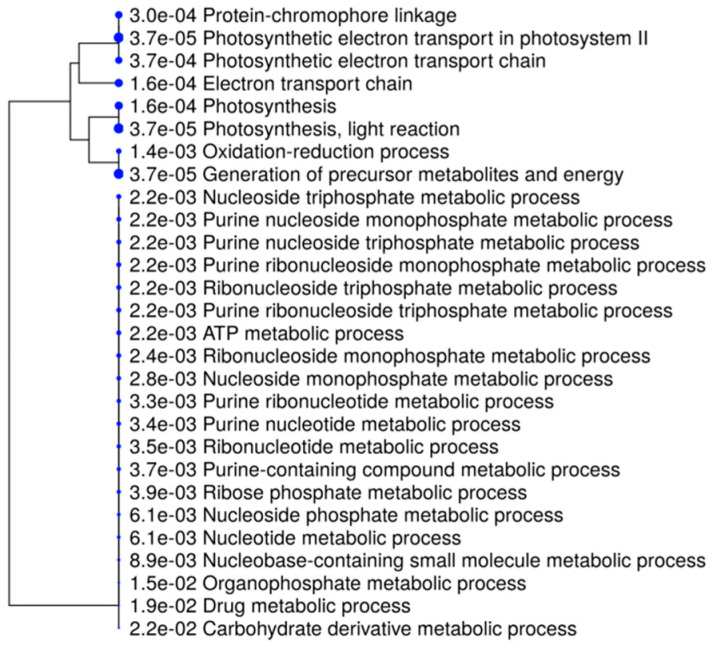
A hierarchical clustering tree. Pathways with many shared genes are clustered together. Larger dots indicate the more highly significant *p*-values.

When the genes specific for each sample were considered, as in the case of Malta 5, they were significantly enriched (*p* < 0.05) for different biological processes, as shown in Figure 7. In particular, those with low significant *p*-values were related to photosynthetic electron transport of photosystem II, photosynthesis, light reaction and generation of precursor metabolites and energy.

## 3. Discussion

As sessile organisms, plants have evolved sophisticated mechanisms of gene regulation that allow them to survive in different environmental conditions, with their extreme fluctuations, as in those related to climate change. Plants must be able to regulate gene expression quickly, in order to adapt to the environmental challenges. This rapid response includes, of course, an efficient sensing of signals, efficient transmission through a cascade of signal transduction pathways and, subsequently, synthesis and mobilization of the relevant transcription factors [10]. At the same time, plant responses to environmental changes and other stresses also involve epigenetic regulation (e.g., modifications of gene expression that occur without changes in the DNA sequence [20]). The main epigenetically driven modifications belong to three types of mechanisms: chromatin restructuring and post-translational modification of histones, DNA methylation changes, and post-transcriptional modifications regulated by non-coding RNAs (microRNA or long non-coding RNAs) [21]. These mechanisms can act synergistically to reinforce the overall chromatin packaging density, which in turn affects the transcriptional state of genes [22]. In plants, DNA methylation is a major epigenetic phenomenon that results in longer-lasting gene expression changes, and the mechanisms controlling DNA methylation inheritance are well established [23,24]. Given the high prevalence and functional implications of 5mC as an epigenetic marker, its detection has been widely investigated. Many experimental approaches have been employed to detect cytosine methylation status in plants [25,26,27]. In general, these methods include the bisulfite conversion, affinity enrichment and restriction-enzyme-mediated filtration, and more recently, next-generation sequencing (NGS). Another commonly employed molecular method for evaluating DNA methylation in the adaptation of non-model plants is MSAP, which is an easy, effective and low-cost approach. In plants, MSAP was used for the first time in rice [28]. After that, the method was employed for examining epigenetic variations in natural populations of various plant species, including herbaceous (viola, orchis, *Dactylorhiza* spp., giant reed, etc.) and woody species such as mangroves and poplars. Recently, the standard MSAP protocol has been enhanced by deep amplicon sequencing using NGS. Baranek et al. (2016) reported for the first time the combination of MSAP standard analysis (digestions of DNA with *Eco*RI and *Hpa*II or *Msp*I enzymes), followed by the NGS of PCR selective amplicons, to study two stable somatic mutants of wheat [29]. Analysing two wheat genotypes in combination with their somaclones, characterized by a changed heritable phenotype, the authors were able to study epigenetically induced changes and, at the same time, identify over 100 amplicons that were differentially methylated among the samples. Another MSAP-based approach combined with NGS is MSAP-Seq [14], which is also integrated with an automatic pipeline MSEQER. This method used conventional MSAP analysis (digestion of DNA with *Eco*RI and *Hpa*II enzymes) and replaced the gel separation of amplicons (from selective PCR) with NGS. To validate the procedure, the authors provided two case studies that measured the effects of different stresses on DNA methylation in barley. One of these studies was focused on the modulation of the leaf methylome in plants grown under water deficiency, followed by drought recovery. The new method allowed the authors to identify around 3000 different DNA sites that underwent methylation changes under water stress—many of which were located within genes or in repetitive elements. The second case study compared barley methylomes of different plant organs (leaf vs. root) during drought and the subsequent re-watering step. The analysis was a validation of the MSAP-Seq method for this kind of analysis, and interestingly revealed that, under stress conditions, some gene regions were subject to transient and reversible methylome modifications, while many repetitive elements underwent irreversible methylation or completely reversible demethylation. Recently, an adaptation of MSAP-Seq has been employed to probe the DNA methylation status in different tissues of *Eucalyptus grandis* W. Hill. and generate a reference genome of the species [30].

In our study, we combined MSAP with NGS to study the DNA methylation status of white poplar monoclonal stands from the island of Malta. We modified the MSAP method by reducing the PCR amplification steps (eliminating the second PCR step) and applying appropriate biostatistical analysis of NGS data. We employed the original MSAP protocol, where genomic DNA is divided into two aliquots and digested with the enzyme *Eco*RI, which has a restriction site that is only negligibly affected by DNA cytosine methylation, and one of two isoschizomers (*Msp*I or *Hpa*II) exhibiting differential sensitivity to cytosine methylation. According to the genome size and abundance of the restriction sites in the DNA, after ligation to two specific DNA adapters, compatible with *Eco*RI- or *Msp*I/*Hpa*II-generated DNA ends, the DNA fragments were PCR amplified. By performing only one round of PCR amplification using pre-selective primers complementary to the adapter sequences, we were able to obtain a very large subset of fragments for the NGS step, whereas with conventional MSAP, only a few hundred (or, in a few cases, thousands) of amplicons are potentially scorable after capillary gel electrophoresis. In addition, performing NGS with MSAP-Seq avoids the formation of homoplastic bands, which is a common problem in gel-based MSAP studies [31]. As a result of these modifications, we were able to survey the entire white poplar genome. Moreover, the method described here is readily adaptable to other non-model organisms.

In our survey, all reads obtained were used to assemble a de novo reference genome for *P. alba*, and we evaluated the quality of the assembly in terms of read representation by mapping reads back to the assembled genome. The quality of the assembly, assessed by the number of contigs generated for both data sets (*Eco*RI-*Msp*I and *Eco*RI-*Hpa*II) was high, and confirmed the reproducibility of the MSAP method, as demonstrated by the good correlation among different clonal samples (e.g., biological replicates, or ramets). When the genome sequence of the species of interest is unavailable, genomic resources such as protein sequences and/or genomic scaffolds from a closely related species serve as reference scaffolds to align and realign de novo assembled contigs. For our analysis, each contig was identified/annotated by alignment against the *Arabidopsis thaliana* genome [32], because this species forms the most prominent basis for annotations for other plant species, more closely related genomes that have been published more recently [33].

The interpretation of MSAP data is based on the recognition of *Msp*I or *Hpa*II restriction sites, which can be modified by methylation, often due to pedoclimatic variations. We performed the comparison of the fragments derived by both digestion patterns, within each single sample and among all the analysed ones. This comparison allowed us to identify the number of unaffected genes (shared genes between *Msp*I and *Hpa*II datasets—M1-H1), and those affected by the DNA methylation (by double-strand methylation of inner cytosine or the hemi-methylation of inner cytosine (M1-H0); or by the hemi-methylation of CHG-sites (M0-H1)). The results obtained suggest that similar methylation polymorphisms were present in the clonal white poplar populations. Regarding the functional annotation, a pathway analysis was performed for each sample and for each DNA methylation status, in order to identify those pathways enriched for genes showing different DNA methylation status, and to discover the potential role of specific DNA sequences in epigenetically-driven processes in white poplar. When a single poplar population/stand was considered, a similar trend in the pathway analysis was observed, revealing a similar influence of the environment on gene regulation, which were mainly involved in chloroplast and mitochondria (essential in fully expanded leaves), photosynthesis, generation of precursor metabolites and energy, and oxidation-reduction processes.

It is not surprising that several genes involved in photosynthesis and electron transport were affected by methylation. These essential metabolic processes [34] require fine regulation in order to respond to natural environments with highly variable and dynamic conditions. The regulation of photosynthesis-related genes is aimed at satisfying plant metabolic demands and, at the same time, avoiding an over-stimulation of the electron transport chain and the generation of harmful reactive oxygen species. Moreover, photosynthetic processes strongly influence plant fitness. Photosynthesis-related processes must be regulated at the level of the individual leaves, and indeed, individual cells and organelles (chloroplasts and mitochondria). Our analyses were performed on fully expanded and photosynthetically active leaves. It is therefore not surprising to find these genes among our results, although their high prevalence was unexpected. It would be interesting to expand our analysis to leaves at different positions in a single tree, in order to investigate these regulatory mechanisms more deeply.

The category “generation of precursor metabolites and energy” includes proteins related to primary metabolism, such as carbon fixation, glycolysis, the citric acid cycle and the electron transport chain. Our results confirmed, once more, that primary metabolism can be generally defined as essential for growth, survival [35] and plant adaptation. Some of the detected DNA fragments were linked to the oxidation-reduction process, indicating that modulation of the cellular redox status is important in changing environmental conditions. Again, our observations highlight the key role of the fine-regulation of gene expression in leaves as paramount for the energy-acquiring photosynthetic process.

Comparisons made between all the samples identified genes with the same DNA methylation status that were specific to individual samples or shared between the five white poplar populations/stands. Among the genes unaffected by methylation, the shared ones (28) within samples were not significantly enriched for any biological process. Meanwhile, among the specific genes per sample, some expression pathways were enriched—for example, biological processes involved in response to viruses and to heat (in the case of Malta 1 population/stand) and those related to mitochondrial translation and mitochondrial gene expression (for Malta 5 population/stand). Mitochondria play a pivotal role in cellular energy metabolism. Specific changes in the rates of respiration are required to meet alterations in energy demands at specific stages in plant growth and development, or under different environmental conditions. The regulation of organelle biogenesis and activity requires numerous nuclear proteins to regulate/modulate transcription, splicing, trimming, editing and translation of organellar RNAs, which requires nucleus-to-organelle (anterograde) communication. Furthermore, all of these processes are mediated by numerous nuclear-encoded cofactors, which are only partially identified and deciphered.

Some genes affected by double-strand methylation of the inner cytosine or hemi-methylation of the inner cytosine were specifically identified in a single poplar population/stand, and were significantly enriched in some biological processes (e.g., those related to metabolic processes (Malta 3 and 5 populations/stands) or to cellular respiration and energy derivation by the oxidation of organic compounds (Malta 5 population/stand) confirming the relevance of epigenetic modulation for metabolism and energy pathways in white poplar.

In the case of hemi-methylated CHG-sites, the genes shared among all the samples were more abundant (19) than the specific ones (for Malta 1, Malta 2, Malta 3, Malta 4 and Malta 5 populations/stands were thirteen, six, two, ten and six, respectively). However, they were not significantly enriched for any biological process, but they included some genes encoding tRNAs. The tRNA molecules contain the most abundant post-transcriptional modifications, and are crucial for proper gene expression and protein biosynthesis. Moradi et al. (2019) found that cucumber plants resistant to the biotic stress caused by the powdery mildew pathogen showed demethylation in those DNA regions located within or near tRNA coding genes [36]. The authors hypothesized that the demethylation of tRNA genes might suppress the expression of genes that are essential for increasing the availability of certain amino acids present in defence proteins.

When the genes specific for each sample were considered, only in the case of the Malta 5 population/stand, pathways related with photosynthesis, light reaction, and the generation of precursor metabolites and energy were enriched. Malta 5 was collected in a more remote site than the others; moreover, from a geographical analysis it emerged that this stand lives in an area characterized by a different lithology (Figure S1D). This is not sufficient to demonstrate the correlation between habitat and epigenetic response, but it suggests that many other features, both ecological and pedo-climatic, should be taken into account.

In summary, this study allowed us to further elaborate the nature of DNA methylation changes for populations and stands of white poplar, the existence of which we observed previously in two different species [8,9,37,38]. To extend these studies, it would be necessary to evaluate the extent and nature of DNA methylation changes not just among individuals of a clonal population in different environments, but also in organs at different positions on the same plant (e.g., leaves). Those differences sustain the hypothesis that DNA methylation modifications can be considered as an epigenetic marker that is strictly related to the habitat where plants live, and to the position of specific organs within the plant.

## 4. Materials and Methods

### 4.1. Plant Material

Well-expanded mature leaves from five white poplar trees were collected from natural populations/stands on the island of Malta (Figure S1A). These samples, which were previously characterized by means of microsatellite markers [15], showed a very limited genetic biodiversity.

### 4.2. DNA Isolation and MSAP Analysis

Total genomic DNA was isolated from leaves (100 mg) using the DNeasy Plant Mini Kit (Qiagen, Hilden, DE). Purified DNA solutions were then processed by means of the MSAP method [12], as described in [8]. Briefly, we used the isoschizomers *Hpa*II and *Msp*I (methylation-sensitive restriction enzymes) as “frequent cutters” and *Eco*RI as a “rare cutter” enzyme (restriction enzyme source: Fermentas, Milano-IT). The different sensitivity of the two isoschizomers *Hpa*II and *Msp*I towards methylation is reported in Table 1, where 1 indicates the presence of fragment and 0 its absence. The fragments were ligated to adapters in order to allow a first PCR amplification (pre-selective). Usually, at this step, the MSAP protocol foresees a second PCR (selective PCR) that is more selective than the first one, due to the use of the same primers but with a longer tail (two/three DNA bp added at the 3′-end tail). The effect of this step is to reduce the number of amplicons, compared to those produced by the first PCR step. Potentially, this leads to a loss of information, but it permits the separation of the DNA fragments in a common automated DNA sequencer. In order to avoid reducing the number of amplicons, in our modified MSAP protocol we sequenced the amplicons that were obtained from the first, pre-selective PCR amplification.

### 4.3. Library Preparation for NGS Analysis of Amplicons

In order to sequence the large number of amplicons produced with the first MSAP-PCR, the following steps were performed. An end-repair step is needed when PCR amplicons contain sticky ends, as in the case of the *Eco*RI restriction sites. Briefly, 32 μL of the fragmented amplicon solution were added to 15 μL of end repair and adenylation buffer and 3 μL of end repair and adenylation enzyme mix (NEXTFLEX, PerkinElmer, UK). This mixture was incubated in a thermocycler under the following conditions: 22 °C for 20 min, 72 °C for 20 min and then 4 °C on hold. Next, five barcoded adapters were ligated to each sample by adding 47.5 μL of ligase enzyme mix (NEXTFLEX, PerkinElmer, Beaconsfield, UK) and 25 ng of DNA barcode adapter to 50 μL of end-repaired and adenylated DNA amplicons. Reactions were incubated in a thermocycler for 15 min at 22 °C. Ligated DNA amplicons were precipitated by adding 0.1 volumes of cold (4 °C) 3 M Sodium Acetate (NaAc) solution and two volumes 96% ethanol (stored at −20 °C) and left at −20 °C overnight. The precipitated DNA amplicons were centrifuged at 14 kRPM for 15 min. DNA pellets were washed with 70% ethanol and mixed in 50 μL of resuspension buffer. The ligation mixtures were used as template for PCR amplification. The PCR amplifications were performed in a final volume of 50 μL, containing 5 μL of ligation mixture and 12 μL of dedicated PCR master mix (NEXTFLEX, PerkinElmer-UK) under the following conditions: initial denaturation at 98 °C, followed by 6 cycles of 98 °C for 30 s, 65 °C for 30 s and 72 °C for 60 s, with a final elongation step at 72 °C for 4 min. Finally, the amplicons were precipitated with 0.1 volumes of 3 M NaAc and two volumes of absolute EtOH, centrifuged at 14 kRPM, and the DNA pellets were mixed in 21 μL of resuspension buffer. For the cluster generation, the libraries were sequenced using the Illumina HiSeq 1500 system (Illumina, San Diego, CA, USA).

### 4.4. NGS Data Analysis

Sequence quality was verified with the UNIX-based package fastQC-0.10.1 software. Reads were processed by CLC suite (CLC/Qiagen, Aarhus, Denmark) in order to filter, remove the unused adapters and polyA, and trim the sequences. Since the sequence of the entire genome of *P. alba* was not available at the time of the study, the reads obtained were analysed through de novo assembly and pooling all the reads relative to each sample (both *Msp*I and *Hpa*II fragments). In this way, a list of contigs was produced. Then, the fragments obtained from each double digestion (*Eco*RI-*Msp*I and *Eco*RI-*Hpa*II) were mapped using these contig lists. The sequences with less than two counts of reads were discarded. In order to check the robustness of the analyses, a Venn diagram was built separately for either *Msp*I or *Hpa*II profiles, considering the ID of the contigs found in each sample.

Contigs of each sample and for both *Msp*I and *Hpa*II fragments were analysed for similarity by BLASTN using as reference genomes those of *Populus trichocarpa* Torr. & A.Gray ex. Hook., *Zea mays* L. (results not shown for both) and *Arabidopsis thaliana* (L.) Heynh., which is also the basis of annotations of the newly published *P. alba* genomes [38].

Subsequently, in order to apply the data interpretation foreseen in the case of the MSAP protocol, consisting of the comparison between both MSAP profiles (*Msp*I and *Hpa*II) of the same analysed sample, a Venn diagram for each sample was elaborated including the genes obtained by *Msp*I digestion vs. those obtained by *Hpa*II digestion. On the basis of the presence or absence of the genes in one category or both categories (*Msp*I and *Hpa*II), the DNA methylation status of the identified genes was estimated as reported in Table 1. In order to identify pathways enriched for genes showing different DNA methylation, gene ontology analyses were performed separately for each sample and for each DNA methylation status, using the Panther classification system [39,40]. Only the results with a false discovery rate (FDR) < 0.05 were considered further.

Furthermore, in order to identify genes showing the same DNA methylation status that were specific for each sample or shared among the five samples, three Venn analyses were generated. The shared genes and the specific ones were also analysed using the ShinyGo [41] web-based software on gene ontology (GO) for annotation and gene ID, mapping plant genomes in Ensembl BioMart release 96.

**Figure 8:**
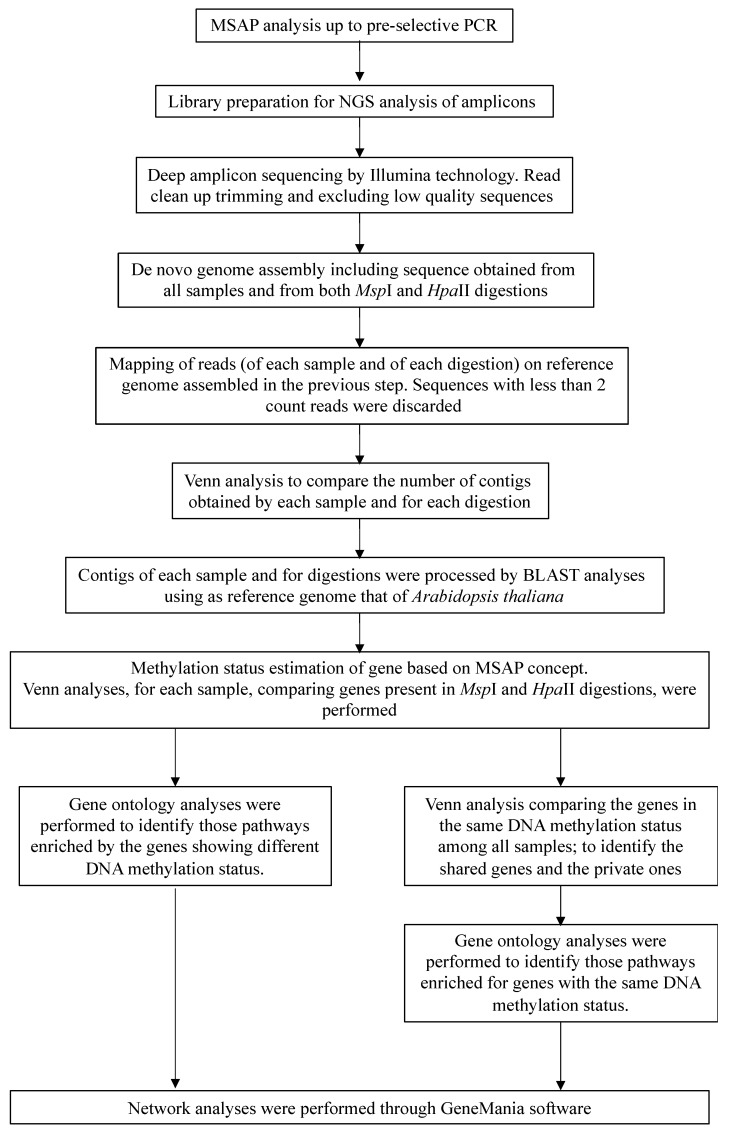
Experimental pipeline of the methylation-sensitive amplified polymorphism (MSAP) and next-generation sequencing (NGS) combined approach.

Network analyses were performed using the GeneMania software [42], which identifies genes related to a set of input genes using a very large set of functional association data. Association data include protein and genetic interactions, pathways, co-expression, co-localization and protein domain similarity. The overall experimental pipeline and approach used for extraction of the final set of sequences are summarized in Figure 8.

## 5. Conclusions

This study on clonal white poplar populations/stands of the Maltese archipelago focused on the integration of two reliable and powerful analytical tools—namely, the cost-effective MSAP analysis and NGS—to identify genes and pathways affected by changes in DNA methylation. This approach is particularly well-suited to plant species for which the genome has not yet been sequenced. Using this combined approach, we showed that it is possible to exploit the NGS technology to sequence the amplicons obtained by the MSAP protocol and to utilize the same biostatistical methods as those employed for the analysis of DNA methylation status revealed by MSAP analysis. Furthermore, it allowed us to identify DNA sequences/genes in which the DNA methylation status was most likely determined by pedoclimatic changes and in response to external stimuli. Our results confirm that this combined approach is reliable and informative and is especially suitable for unannotated genomes.

## Figures and Tables

**Table 1:** Restriction enzyme behaviour: *Msp*I and *Hpa*II sensitivity to methylation at cytosines in their digestion site.

*HpaII*	*MspI*	Methylation Status
1	1	No methylation
1	0	Hemi-methylated CHG-sites (hemi-methylation of inner and outer cytosine)
0	1	Double-strand methylation of inner cytosine or hemi-methylation of inner cytosine
0	0	Un-informative state caused either by different types of methylation or due to restriction site polymorphism

## Supplementary Materials

Supplementary Materials can be found at https://www.mdpi.com/1422-0067/21/19/7393/s1.
