# Supplementary material for: Epigenetic Analysis through MSAP-NGS Coupled Technology: The Case Study of White Poplar Monoclonal Populations/Stands
Source: Int J Mol Sci. 2020 Oct 7;21(19):7393. doi: 10.3390/ijms21197393 (PMC7582538; doi:10.3390/ijms21197393)
Supplement: Supplementary file 1 [file ijms-21-07393-s001.zip › ijms-934278-supl-proofed/Figure S1A.pdf]

A

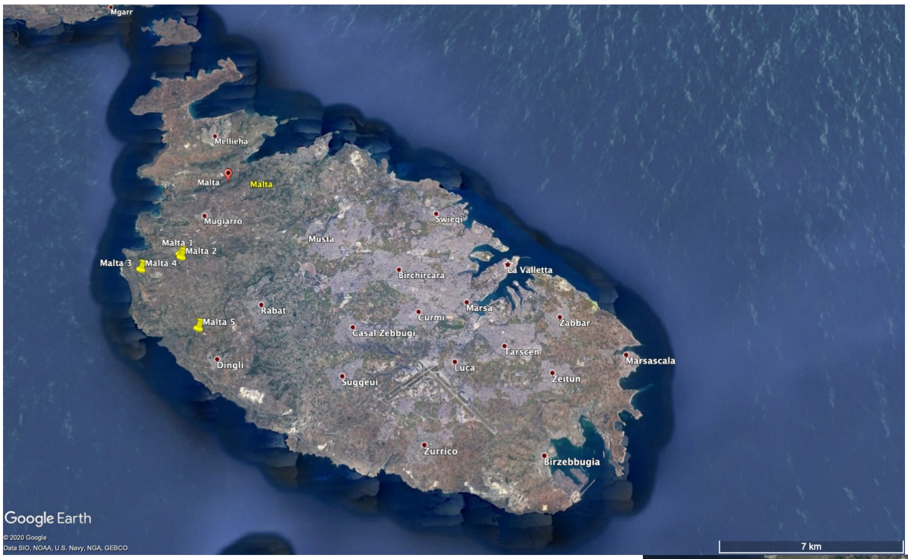

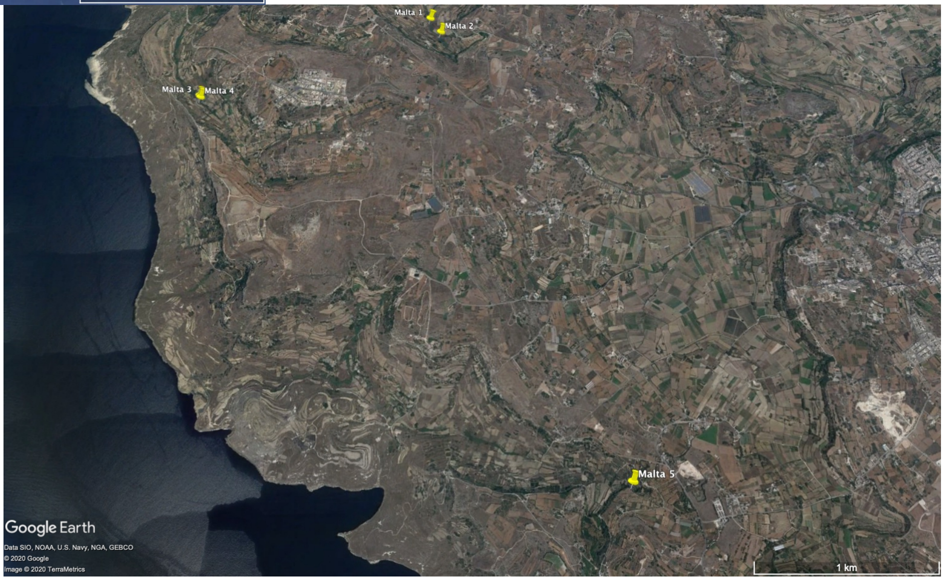

Figure SI 1. Geolocalization of the investigated white poplar trees, in yellow the placeholder where poplars were collected. A) Overview of Malta Island; B) Detail of the area where poplars were collected

Name	Latitude	Longitude	Altitude asl (m)
Malta 1	35.900268	14.356678	254
Malta 2	35.899025	14.357558	178
Malta 3	35.894149	14.338954	144
Malta 4	35.894036	14.339035	144
Malta 5	35.870093	14.370083	230
