# Supplementary material for: Epigenetic Analysis through MSAP-NGS Coupled Technology: The Case Study of White Poplar Monoclonal Populations/Stands
Source: Int J Mol Sci. 2020 Oct 7;21(19):7393. doi: 10.3390/ijms21197393 (PMC7582538; doi:10.3390/ijms21197393)
Supplement: Supplementary file 1 [file ijms-21-07393-s001.zip › ijms-934278-supl-proofed/Figure S1D.pdf]

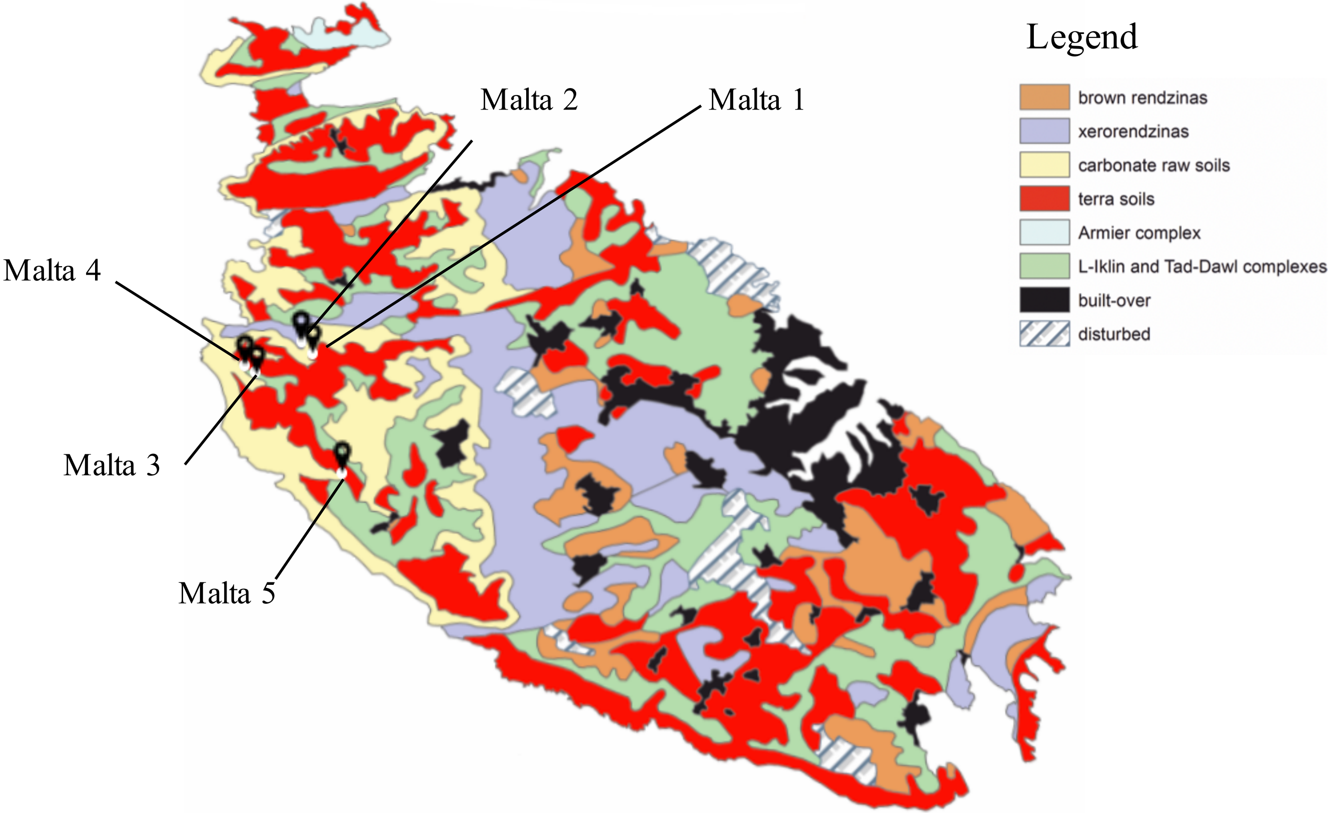

Malta 2
Malta 1
Malta 4
Malta 3
Malta 5
Legend
brown rendzinas
xerorendzinas
carbonate raw soils
terra soils
Armier complex
L-Iklin and Tad-Dawl complexes
built-over
disturbed
