# Supplementary figures and images for: Epigenetic Analysis through MSAP-NGS Coupled Technology: The Case Study of White Poplar Monoclonal Populations/Stands
Source: Int J Mol Sci. 2020 Oct 7;21(19):7393. doi: 10.3390/ijms21197393 (PMC7582538; doi:10.3390/ijms21197393)

Distribution by gene type  
Chi-squared test  $P = 3.5E-10$  \*\*\*

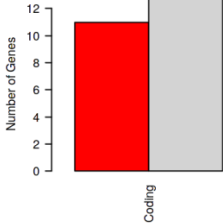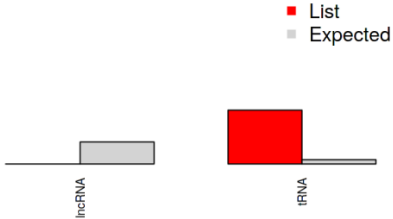

Supplement: Supplementary file 1 [file ijms-21-07393-s001.zip › ijms-934278-supl-proofed/Figure S1B.pdf]

Distribution by gene type  
Chi-squared test  $P=1.3E-167$  \*\*\*

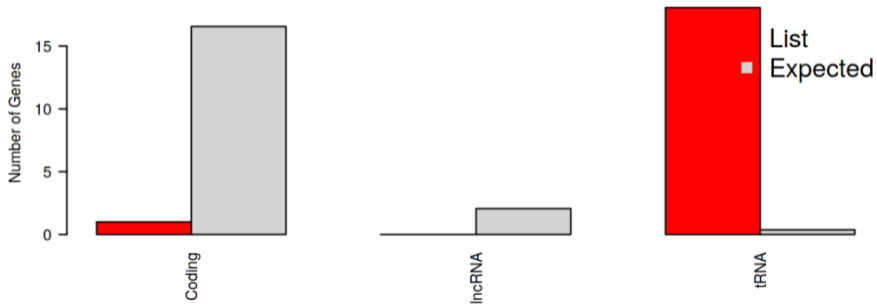

Supplement: Supplementary file 1 [file ijms-21-07393-s001.zip › ijms-934278-supl-proofed/Figure S1C.pdf]
